# Two Interesting Results from the Use of Pituitary Extract

**Published:** 1912-09

**Authors:** W. G. Doern

**Affiliations:** Formerly Professor of Anatomy and Principles of Surgery, Milwaukee Medical College. Attending Surgeon St. Joseph’s Hospital. Milwaukee, Wis.; 415 Grand Ave.


### Two Interesting Results from the Use of Pituitary Extract.

By W. G. DOERN, M.D.
Formerly Professor of Anatomy and Principles of Surgery, Milwaukee Medical College.
Attending Surgeon St. Joseph's Hospital.
Milwaukee, Wis.

WE now find animal therapy more thoroughly established, than ever before, in present day treatment by the advanced medical men of the country. While many of the theories advanced as to the therapeutic value of some of these agents are yet problematical, many of them have been very thoroughly established by the vast preponderance of evidences in clinical results.

Although the future of this class of agents is promising, it will require no little time and mass of clinical reports to prove or disprove the many theories that have already been advanced.

One of the most recent subjects of investigation is the extract of the Pituitary body. This little organ snugly hidden away at the base of the brain has long been and still is an enigma to science. Some of our early research workers concluded that it had no purpose other than a cushion or protection for the brain. Modern science long since ceased to doubt that the Pituitary body was of vital importance to the functions of certain other of the human organs and it has been shown by experiments that there is a relationship between the thyroid gland and the Pituitary body and a possible relation to that of the suprarenal glands. Just what their relationship is becomes a matter of considerable speculation. The fact that the organ is situated so remote in the human anatomy far from any field of observation renders the possibility of human experiments almost impossible. Our knowledge is therefore based upon animal experimentation and such clinical observations as have been possible to make.

It has been my privilege to observe two very interesting cases, treated with an extract of the infundibular body which was prepared by the Department of Experimental Medicine of Parke, Davis & Co. My purpose in reporting these cases is to add just so much to the information which is so eagerly sought by those who are interested in the scientific advancement of modern medicine, with the hope of inviting a co-operation from other medical men who have had as much or perhaps more experiences with the Pituitary Extract.

Case No. 1—Miss G. Seamstress. Age 26. History; severe headaches continuing for weeks without relief. While the pain was general through the head, it was usually worse on the right side. At times there was noticeable swelling or puffing of the eye lids. The refraction was apparently normal, and there was no objective or subjective symptoms that would indicate the real cause of the condition. Anodyne and sedative treatment afforded temporary relief and that for only a short time. A general physical examination revealed nothing of a positive nature, except that the patient would pass about 1½ pints of urine in 24 hours, specific gravity, 1.008. There was no evidence of inflammatory condition of the kidney other than a real insufficiency.

As an experiment, this patient was given 10 drops of Pituitary Extract 3 times a day and within 3 days the amount of urine was increased from 1½ to 2½ pints in 24 hours. With this came a noticeable improvement in the condition of the patient, with practically a complete relief from all headache and eye conditions.

Case No. 2—Mr. B. Age 52. Married. Occupation, Business Manager of a large manufacturing concern. History:—has always been strong and vigorous with no severe illness during his entire lifetime. For about 6 months previous to the present time, he had noticed a lack of potentia sexualis, otherwise apparently in perfect health. He was placed on a treatment of 15 drops of the Pituitary Extract 3 times a day with no other treatment. In a short time, there was a noticeable improvement in his condition and at the end of 3 weeks, the potential energies were restored to normal. This patient had reported several times during the past 3 months and each time has shown permanent benefit without a continuation of the treatment.

This medication suggested itself to me owing to the marked stimulating effect which the Pituitary substance has upon the sexual organs, as indicated by the lack of sexual development or loss of powers in cases of tumors of the Pituitary Body.

I trust that the report of these cases will stimulate other physicians to report similar cases should they by chance have had the opportunity to observe them.

415 Grand Ave.

